# Late Ordovician Mass Extinction: Earth, fire and ice

**DOI:** 10.1093/nsr/nwad319

**Published:** 2023-12-18

**Authors:** David A T Harper

**Affiliations:** Palaeoecosystems Group, Department of Earth Sciences, Durham University, Durham DH1 3LE, UK

**Keywords:** Ordovician, LOME, paleoecology, palaeobiogeography, biotas

## Abstract

The Late Ordovician Mass Extinction was the earliest of the ‘big’ five extinction events and the earliest to affect the trajectory of metazoan life. Two phases have been identified near the start of the Hirnantian period and in the middle. It was a massive taxonomic extinction, a weak phylogenetic extinction and a relatively benign ecological extinction. A rapid cooling, triggering a major ice age that reduced the temperature of surface waters, prompted a drop in sea level of some 100 m and introduced toxic bottom waters onto the shelves. These symptoms of more fundamental planetary processes have been associated with a range of factors with an underlying driver identified as volcanicity. Volcanic eruptions, and other products, may have extended back in time to at least the Sandbian and early Katian, suggesting the extinctions were more protracted and influential than hitherto documented.

## Teaser

The Late Ordovician mass extinction was the first of the big five Phanerozoic extinctions and the first to affect animal life in oceans. The extinctions removed about 75% of animal species and were coincident with a major ice age. The search for an ultimate cause has proved difficult for both the extinctions and ice age, but accumulating evidence points to a possible volcanic trigger.

## INTRODUCTION

The Late Ordovician Mass Extinction (LOME) was the earliest of the five ‘big’ extinction events and the earliest to involve metazoan life. Many have described the extinctions, apparently related to the waxing and waning of Gondwanan ice sheets, as a conundrum or a paradox [1 and references therein], since major biodiversity loss elsewhere in the Phanerozoic is not intimately associated with ice ages. Few, however, would doubt the coincidence of the Late Ordovician extinctions with a major ice age. Many if not all of the extinctions were related to habitat disruption and destruction as consequential symptoms of the properties of this icehouse world and its immediate aftermath. There have been many excellent reviews of these events, and these are referred to and in places summarized here. The main purpose of this review is to highlight a number of the key issues and our current understanding of this extinction, its commonality with others and its own unique facets.

## BROADER CONTEXT

The concept of mass extinctions, when over 50% of species disappear from Earth over a short time interval, has been known for over 200 years. The idea of extinction was first introduced by the English polymath and science officer in the Royal Society, Robert Hooke (1635–1703) in the late 1600s, but it was the French comparative anatomist and palaeontologist, Baron Georges Cuvier (1769–1832) who identified intervals of mass extinction in the Cenozoic rocks of the Paris Basin during the late 1700s within mammal and mollusc faunas. Later, during the early 1800s recognition of a series of biotic turnovers, commonly associated with unconformities, helped form the basis for our identification of the different geological systems across Europe. Cuvier, however, pointed out that two events were particularly marked: the end Permian and the end Cretaceous. These intervals of rapid and profound biological change partitioned the Phanerozoic into the Palaeozoic, Mesozoic and Cenozoic eras. These intervals were particularly obvious when portrayed on John Phillips's diversity curve for the Phanerozoic (1860), the first based on actual fossil range data, and allowed a more formal approach to the definition of these erathems. Significantly the mass extinctions at the end of the Ordovician (LOME) were hidden by the fact that Roderick Murchison's Silurian System contained his Lower and Upper Silurian divisions in a presumed uninterrupted geological period within the ‘Greywacke’; the former is now assigned to the Ordovician based on its recognition by Charles Lapworth (1842–1920) developed through his understanding of the differences between the Hartfell (Ordovician) and Birkhill (Silurian) graptolite faunas in the Scottish Southern Uplands [2]. Ironically changes in these graptolite faunas had already signaled a major extinction.

Species become extinct when their entire interbreeding population is eliminated thus generally speaking, small, localized populations are more susceptible to extinction than widespread, abundant species. Local populations, particularly those with limited geographical ranges, if they are unable to adapt or migrate, can be wiped out by habitat disruption and destruction, severe climatic events, and the introduction of new diseases, parasites or predators. In general terms large species and those with specialized diets seem to be more prone to extinction. Many Holocene species have become extinct as a result of habitat disruption on island environments and in tropical rainforests or coral reefs; the Mauritius dodo, New Zealand moa, the South African quagga, the Tasmanian tiger and the North American passenger pigeon, for example, were hunted by humans to extinction whereas the endemic Christmas Island rats were fatally infected by pathogens carried by fleas hosted by invasive black rats.

The geological record is generally characterized by low levels of background extinction; the average longevity of a species is generally ∼1 myr for mammals and 11 myr for invertebrates [3]. However, a number of intervals are marked by the removal of an unusually high number of taxa from a wide variety of both clades and habitats during a relatively short period of time. Today, with the notable exception of the end-Cretaceous meteorite impact, the major mass extinctions are associated with terrestrial agents such ice ages and volcanicity; the extinctions proceeding during the Late Holocene are associated with human activity. The concept of mass extinction associated with extra-terrestrial factors, though attractive, had few advocates until the 1950s when, for example, Norman Newell (1909–2005) and Otto Schindewolf (1896–1971) began to emphasize both the periodicity of mass extinction and the need for extra-terrestrial causes, based on their compilations of data. Nevertheless, it was not until the late 1970s to early 1980s that the causes and consequences of mass extinctions were scientifically framed and taken seriously by the palaeontology community as a whole.

**Figure 1.**
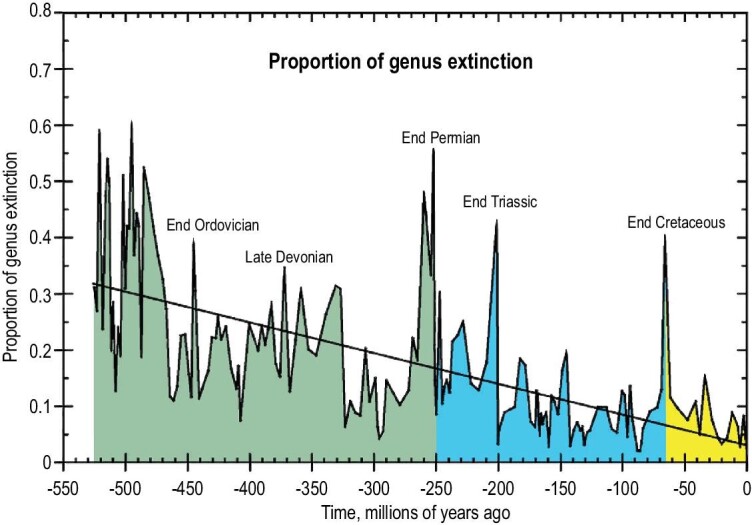
Proportion of generic extinction during the Phanerozoic, highlighting the big five extinction events (redrawn from [97]).

These mass extinctions, removing over 50% of existing species, having short durations and a widespread geographical reach, have occurred several times during the last 550 myr (Fig. 1). Traditionally five ‘big’ extinctions have been recognized (see below). Recent analyses, however, suggest that only three, the terminal Ordovician, terminal Permian and terminal Cretaceous were exceptional [4]; the largest by far was the terminal Permian. The causes for the terminal Devonian [5], terminal Permian [6 and references therein], end Triassic [7] and terminal Cretaceous [8] extinctions are now well established. The first three are associated with habitat restriction and loss in greenhouse worlds driven by volcanicity; the fourth is associated by a meteorite strike involving habitat destruction but also the killing of organisms as a direct result of the meteorite strike.

The Ordovician in particular represents a conundrum, being coincident with the waxing and waning of ice sheets during major glaciation events. This is problematic since atmospheric CO_2_ levels were apparently high and the more recent ice ages such as those in the Pleistocene are not associated with mass extinctions. No one cause can account for all the end Ordovician extinctions but volcancity during the Late Ordovician is emerging as a unifying factor for the causes. But the effects were profound. In many parts of the world sea levels fell, associated with a transition from deep-water to shallow-water environments, with in places unconformities, and from deep-water *Foliomena* faunas to the more widespread relatively shallow-water *Hirnantia* faunas, changes particularly obvious in Scotland [9] and South China [10].

## THE END OF THE ORDOVICIAN

**Figure 2.**
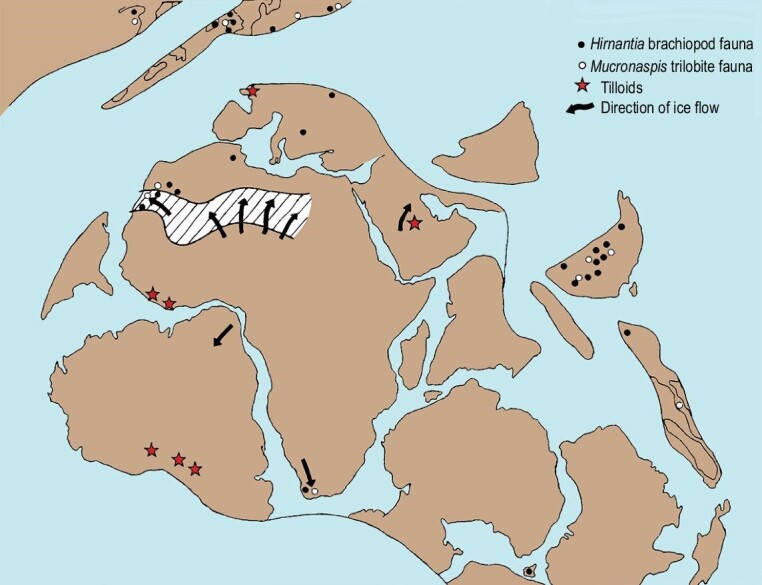
Palaeogeographic reconstruction of Gondwana and its marginal areas during the Hirnantian. Directions of ice movement together with location of key faunas are indicated (modified and redrawn from [18]).

The Late Ordovician Mass Extinction (LOME) was the earliest of the five great Phanerozoic mass extinctions and the first to globally devastate animals and their ecosystems [11,12]. Ordovician climate change was discussed in detail by Spjeldnæs [13,14] and climatic zones plotted on pre-drift palaeogeographic maps. An end Ordovician glaciation, based on glacial deposits in North Africa was noted by Beuf *et al.* [15] and related to extinctions by Berry and Boucot [16] and Sheehan [17]. These latter authors also mapped a clear distinction between Ordovician and Silurian faunas, separated by a major extinction, and related to an episode of global cooling. The track of the ice sheets and the distribution of Hirnantian faunas was first plotted by Cocks and Fortey [18] for near-field Gondwana and its margins (Fig. 2) following a warm phase—the Boda Warming Event [19]. The presence and scale of the ice sheets are now well established [20]. There have been many detailed reviews of the extinctions, focused on various aspects of these events, including data on biotic and environmental changes [11,12,21]. New information is constantly being published to frame and support new and existing hypotheses. There remain, however, a number of key issues:

1. The age and duration of the LOME, one phase or many over a short or longer duration.
2. The main categories of organisms affected within the oceanic and terrestrial realms.
3. Changes in the environment that facilitated or triggered habitat destruction or restriction, biotic migrations and eventual extinction.
4. The main agents of environmental change both globally and regionally.
5. The overarching cause or causes of these environmental changes.
6. Significance for subsequent life in terms of changes in taxonomic diversity and the structure and composition of ecosystems.

### Age and duration

**Figure 3.**
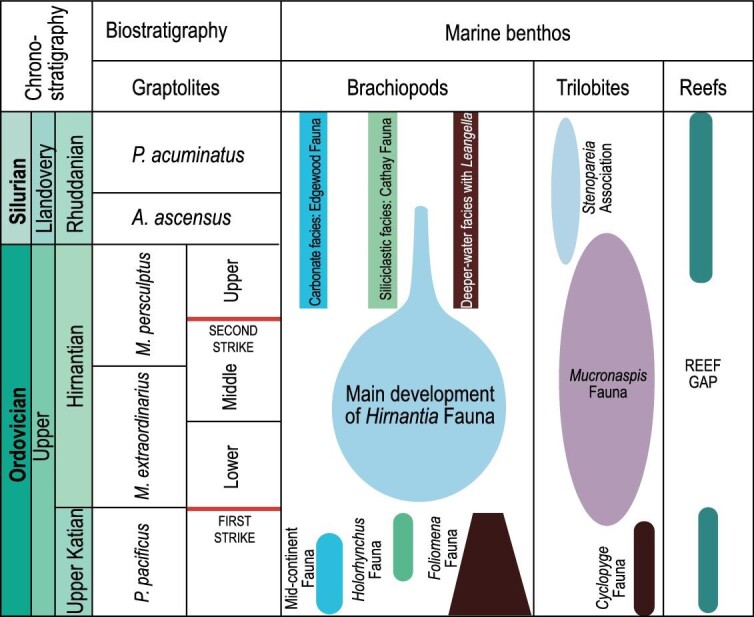
Stratigraphic framework for extinctions during the Late Ordovician, showing chronostratigraphy, graptolite zones, the first and second phases are indicated together with important shelly faunas. Low-diversity *Hirnantia* faunas are recorded from refugia in the highest Katian and the lowermost Rhuddanian (modified and redrawn from [12,37]).

Previous estimates of an extended glaciation from the early Katian into the Wenlock were challenged by Brenchley *et al.* [22], who restricted the main glacial event and its extinctions to the Hirnantian Stage, reducing the duration of the event from 35 to 0.5–1 myr. New radioisotope dating also constrains the glacial maximum of the Hirnantian Stage even further to ∼0.2 myr, supporting its brevity and intensity that probably triggered the LOME [23]. Most authorities agree that the LOME occurred ∼445–443 Ma with recent duration of the entire age at ∼1.4 myr. There are a number of models. First, two main extinction pulses have been detected, mainly on the basis of the stratigraphic distribution of fossils [12], much of which was established during the search for a Global Boundary Stratotype Section and Point for the base of the Silurian System [24]. Many families of brachiopods, graptolites, echinoderms, molluscs, ostracodes and trilobites together with a majority of reef-building animals died out during a first strike. A second strike coincident with a major transgression in the middle Hirnantian was geographically more extensive but less severe [12,25]. These biotic extinctions are coincident with the geological and geochemical evidence for substantial and sudden climatic changes at the termination of the Ordovician and start of the Silurian. Three glacial advances and intervening interglacials have been detected within this framework (Fig. 3). Second, the extinctions have been captured in a single major phase. Wang *et al.* [26] considered the extinctions as part of a single more prolonged phase including the main basal Hirnantian extinction and subsequent survival and recovery phases, the last during the late Hirnantian. Third, the extinction may have been even more extensive including an earlier phase in the early Katian prior to the concluding two phases in the Hirnantian [1,27].

### The stratigraphy of the Late Ordovician Mass Extinction

As noted above, the global search for the basal stratotype for the Silurian System, its Global Boundary Stratotype Section and Point [24], determined the stratigraphy and the range of biotas across the boundary in considerable detail [see also 28 for boundary definition], allowing a more secure and precise correlation of sedimentary strata across the Ordovician-Silurian boundary (Fig. 3). The Hirnantian Stage is subdivided into the lower, middle and upper parts, corresponding, respectively, to the lower-middle *Metabolograptus extraordinarius* biozone, upper *M. extraordinarius* to lower *Metabolograptus persculptus* biozones, and upper *M. persculptus* biozone [11,27–31]. The glaciation reached its acme during the lower-middle Hirnantian, followed by a dramatic sea-level rise climaxed within the upper part, associated with black shales in many parts of the world, where biological productivity may have been aided by nutrient-laden sediments from melting ice. The three parts show distinguishable brachiopod associations, with variants of the *Hirnantia* brachiopod fauna occurring across a range of depths in the lower-middle parts, and the Edgewood-Cathay Fauna [32] in the upper part, following the second extinction pulse.

## BIOTIC TURNOVERS: MAIN GROUPS OF ORGANISMS AFFECTED

**Figure 4.**
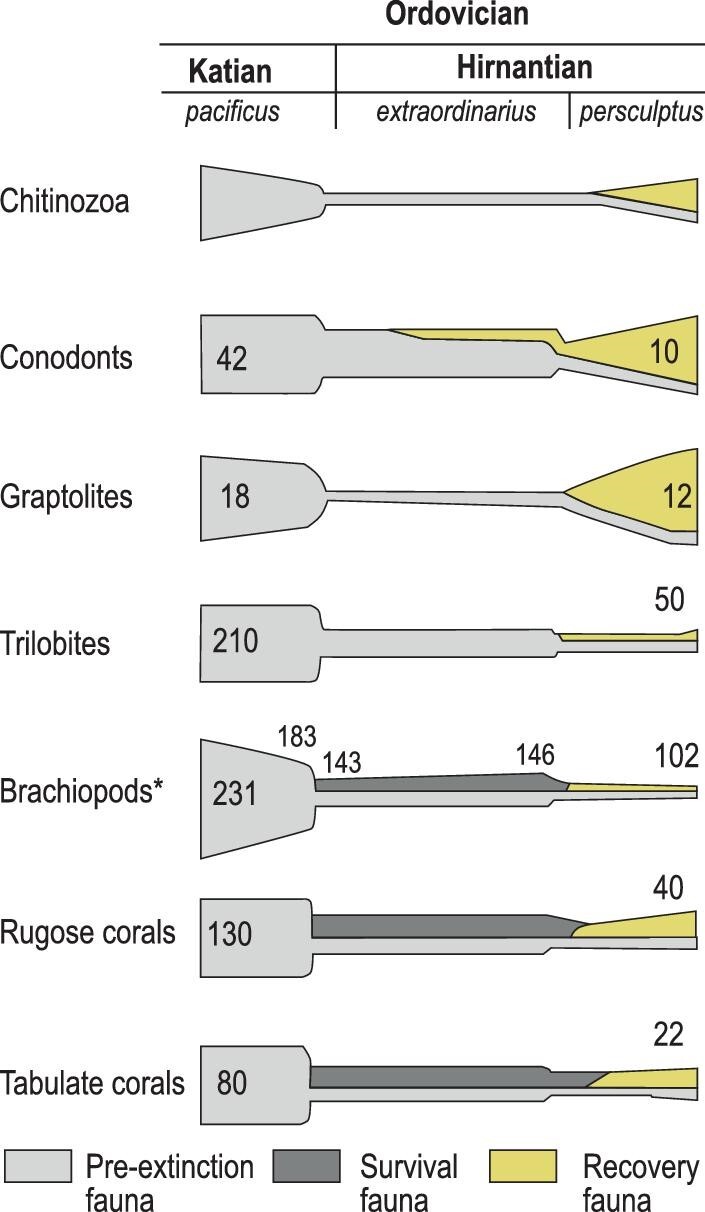
Biotic change through the crisis intervals (from [25] with modifications). *Note also these data indicate the generic loss in brachiopod numbers that appears to have been initiated already within the pacificus Biozone.

The LOME was a major taxonomic extinction but a less severe phylogenetic extinction with most higher orders continuing into the Silurian. The impact on ecosystems was fairly minimal. The substantial amount of biotic data, at alpha, beta and gamma scales, has indicated two clear and well-defined extinction intervals, corresponding to the base and the middle of the Hirnantian Stage, respectively [11 for review]. Data are available in the Palaeobiology Database (PDBD), the GeoBiodiversity Database (GBDB) and many high quality, long-tailed datasets. There have been a number of recent studies [33] on the trajectory of global biodiversity through the Ordovician and a wealth of data is also available [34] for all the major taxonomic groups. These compiled data revealed the eradication of some 25% of families, 40% of genera and 80% of species, witnessing the impact on biotas in different ways and in varying degrees of intensity and the abrupt end in the biodiversity rise known as the great Ordovician Biodiversification Event (GOBE). Microorganisms including phyto- and zooplankton declined in diversity during the first pulse, in association with massive losses of mobile and sessile benthic animals (Fig. 4). The fixed benthos suffered from a much more severe extinction than the mobile benthos, with the pelagic fauna also being particularly targeted [35], showing the variable degree of extinction across the phyla and the variable amounts of diachronism. The biotic data, largely based on Webby *et al.* [34] and the Paleobiology Database, have been reviewed a number of times [i.e. 11,33,36], and in an encyclopaedia entry [37]. These data are thus only briefly reviewed here but their inclusion, together with new data compilations from the Paleobiology Database [38–40], provide the basis for the subsequent narrative.

### Phyto- and zooplankton

Diversity data are sparse for these groups that formed the base of the Ordovician trophic chain. They are not visible to the naked eye, their investigation requires specialist extraction techniques, the use of microscopy and can be restricted to particular facies. The acritarchs reached a peak of some 100 species at the Lower-Middle Ordovician boundary but were reduced in species numbers during the Hirnantian [40]. The chitinozoans tracked the diversity increase of the acritarchs, reaching a peak in the Late Ordovician, but suffered major losses during the latest Ordovician [40]. The diversity of the radiolarians is less well documented and few species are reported; species numbers crashed during the Hirnantian [38].

### Porifera and Cnidaria

Sponges, including stromatoporoids, were key members of the Ordovician benthos populating reef complexes, together with corals, from the Middle Ordovician onwards, in preference to a dominance by micro-organisms [41]. The sponges *sensu stricto* and the stromatoporoids both had peaks in the Middle and Late Ordovician [38] but suffered during the Hirnantian Age. Sponges again peaked following the end Ordovician extinctions [42,43], indicating a phase of recovery, first by simpler organisms [44].

Rugose and tabulate corals diversified during the Ordovician radiation, possibly deriving independently from different soft-bodied cnidarian progenitors deep in the Cambrian Period or even before. The corals became a dominant reef-building group providing a range of ecological niches for other organisms during a Middle Ordovician revolution in carbonate substrates and a following Late Ordovician diversity peak [38]. The two phases of the LOME realigned the relative dominance of the two groups, with the solitary and colonial rugosans becoming the more dominant, particularly in carbonate environments, throughout the remainder of the Palaeozoic Era until their extinction at the termination of the Permian Period [45].

### Lophotrochozoans

The brachiopods are critical in tracing the deterioration of environmental conditions during this biotic crisis due to their dominance of the Ordovician suspension-feeding benthos. These animals show a two-phase extinction in the lower (base of the *Metabolograptus extraordinarius*) and upper (*M. persculptus*) Hirnantian, with the first being more severe [12,32]. A similar situation was also found in rugose and tabulate corals. Nearly 60% of genera of Brachiopoda were eradicated, with 40% being wiped out during the first phase in particular, with the Rhynchonelliformea or articulated brachiopods being most affected [12,32]. The faunal taxa inhabiting relatively shallow-water environments were most effectively removed, and the *Foliomena* fauna in the deep sea, with low-diversity but widespread, small, thin-shelled features was entirely eradicated [32,46]

**Figure 5.**
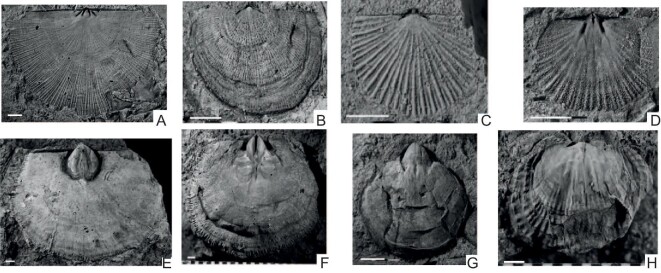
Some of the core genera of the *Hirnantia* brachiopod fauna (Kosov Province), from South China (all internal moulds). (a) *Eostropheodonta* (dorsal valve), (b) *Paromalomena* (dorsal valve), (c) *Fardenia* (dorsal valve), (d) *Dalmanella* (dorsal valve), (e and f) *Hirnantia* (ventral and dorsal valves), (g) *Hindella* (ventral valve) and (h) *Cliftonia* (dorsal valve). Scale bars = 2 mm. Photos courtesy of Professor Bing Huang.

The lower part of the Hirnantian Stage domiciled the near-cosmopolitan *Hirnantia* brachiopod fauna [47] which is believed to be one of the most distinctive, widely-distributed and short-lived animal assemblages of the Phanerozoic marine setting [32], being distinguished by about half a dozen signature taxa (Fig. 5). The fauna shows a rapid expansion after the first crisis in brachiopod global distributions, the geographic occupation from shallow to deep water, and inhabiting mainly mid-shelf siliciclastic but occasionally carbonate environments. During the late Hirnantian, this fauna was replaced by the Edgewood Fauna in carbonate environments and by the Cathay Fauna in siliciclastic environments [32].

Within the rhynchonelliformeans there was a loss of another 30% of the genera during this phase. However, both the Ordovician and Silurian brachiopod-dominated communities show comparable ecological architecture and community structures though they are different in taxonomic composition due to the eradication of large numbers of genera and species. All the same ecological niches were present, for example infaunal, attached epifaunal, recumbent epifaunal, but the roles were occupied by different actors [48].

The cryptostomes and the trepostomes [49], the two main groups of Ordovician Bryozoa peaked during the Late Ordovician but lost ∼15% diversity at the family level during the LOME [12,37]; these two groups remained important but never returned to their former diversity during the subsequent Palaeozoic, their place taken by first the fenestrates and much later in the Mesozoic by the cyclostomes. The succeeding bryozoan faunas in the Llandovery (early Silurian) are as a whole less diverse, and radiated during the later Llandovery and early Wenlock (middle Silurian) to form important parts of the sessile benthos.

The diversity of bivalve molluscs was substantially diminished in nearshore environments during the Hirnantian. Their offshore migration during the early stages of the GOBE was curbed by the two extinction phases, with recovery being retarded. The orthoconic cephalopods as the apex predators at that time were significantly reduced from nearly 150 species at the peak of the GOBE to some 50 species during the Hirnantian [38,50]. This slump in predator diversity was assigned to the destruction of the diversity of habitats for potential prey due to the early Hirnantian regression causing the draining of many epicontinental seas.

### Ecdysozoans

The trilobites were the dominant members of the Ordovician mobile benthos [51]. Some 70% were excised during the Hirnantian. Focusing on the principal feeding strategies, the plankton feeders, predators or scavengers, filter chamber feeders and particle feeders, of those the filter chamber feeders (e.g. the trinucleids) were particularly exposed to extinction. The trilobites inhabiting the deeper shelf were reduced in diversity, together with the mesopelagic taxa, in particular those with reduced eyes or blind forms. In general, the more cosmopolitan species in mid-shelf environments, rather than the more endemic forms, survived the first extinction phase, a pattern obvious in other groups [51]. The major groups such as the mesopelagic cyclopygids were not replaced in the recovery interval, and their place in the oceans was probably occupied by non-trilobite taxa [12].

There was, however, a companion to the near-cosmopolitan *Hirnantia* brachiopod fauna, the widely-distributed *Mucronapsis* trilobite fauna [52]. Together with the *Hirnantia* fauna, that fauna disappeared almost entirely during the second extinction pulse, at the end of the Ordovician Period, surviving in only a few deep-water refugia for a short period of time.

The other main group of arthropods, the ostracodes, suffered comparable substantial losses during the two extinction pulses, with at least 30% of families and 50% of genera gone by the Silurian [12,37].

### Deuterostomes

The declining Katian graptolite fauna was reduced to only a few genera and less than 20 species by the first extinction pulse [35,38]. There was a significant culling of biserial morphologies, for example the dicellograptids, glyptograptids and diplograptids, exhibited a reduction of 65% species diversity. Recovery was swift during the *Metabolograptus persculptus* Biozone. Many adaptive strategies that found success in the Ordovician were reprised during the Silurian in the monograptids, together with bizarre, curved spiral morphologies. Over 200 species were reported from Upper Llandovery strata (Lower Silurian). The extinction and recovery interval has been identified with the greatest precision from the Yangtze Platform of China, where a refugium was documented during the extinction pulses providing a focus for the re-invigoration of the group [53].

Echinoderms were substantially affected with ∼70% of crinoid families disappearing during the two extinction phases, whereas few cystoid and edrioasteroid groups survived the LOME [54]. In fact, sessile crinoid and cystoid echinoderms suffered similar fates at the base of the Hirnantian Stage. Multiple new taxa appeared within the tropical Edgewood Province (see above) during the later Hirnantian, and the echinoderms rapidly recovered in richness during the Llandovery (early Silurian) when they became key players across carbonate substrates.

Conodonts suffered a similar twin-phase extinction [38], with the loss in the order of 80% at the base of the Hirnantian but apparently less pronounced during the second phase. Many new taxa originated, however, during the *M. persculptus* Biozone, migrating from deeper-water environments to inhabit shallow-water communities probably preying on the diversifying fish faunas. There is, however, insufficient data to test the distribution of fishes through the extinction phases; the major expansion of this group occurred in the Silurian and Devonian periods [55].

## ENVIRONMENTAL CHANGES

**Figure 6.**
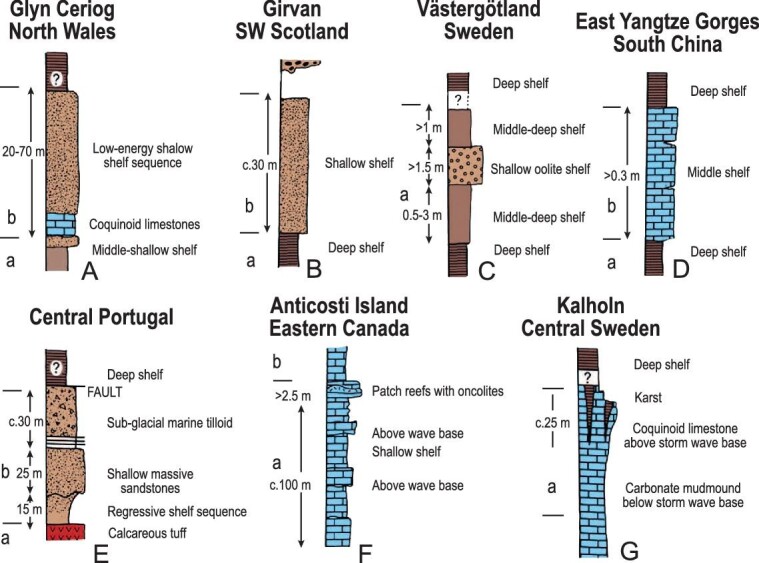
Schematic sections displaying the extinctions and succession of environmental changes around the Ordovician-Silurian boundary in different marine settings (modified and redrawn from [56]). Formations indicated for sections A–G as follows: A. (a) Dolhir Formation; (b) Glyn Formation. B. (a) South Threave Formation; (b) High Mains Formation. C. (a) Dalmanitina Beds; D. (a) Wufeng Formation; (b) Kuanyinchiao Formation. E. (a) Porto de Santa Anna Formation; (b) Ribeira do Braçal Formation; (c) Ribeiro Cimeria Formation. F. (a) Ellis Bay Formation; (b) Becscie Formation; G. (a) Boda Limestone.

The coincidence of the extinctions with the waxing and waning of extensive ice sheets during the terminal Ordovician ice age has been known for half-a-century [16,17]. Changes in facies across the globe in different settings, were profound. Brenchley presented, diagrammatically [56], the effects of a major glacial advance and an associated regression on a range of different geological settings (Fig. 6).

Transitions within shelfal environments at various depths provided critical evidence of the regression. On Avalonia, at Glyn Ceriog (Fig. 6A) mid-shallow shelf sediments are replaced by first coquinoid limestones and second a low-energy shallow water sequence. On the Laurentian margin at Girvan (Fig. 6B), deep-water mudstones give way to shallow-water sandstones, whereas on Baltica in Västergötland (Fig. 6C), deep-shelf sediments are replaced by a variety of mid-shallow shelf deposits. On the East Yangtze Platform (Fig. 6D) deep-shelf graptolite shales are replaced by shelfal limestones. Near the South Pole in Portugal (Fig. 6E) there are shallow-water sandstones and glacigene strata. Farther afield carbonate deposits on Anticosti Island (Fig. 6F) and mudmounds in Central Sweden (Fig. 6G) have disconformities in the shallower-water parts of the succession. These and other successions, including the karstic development at Meifod in mid-Wales, provide critical evidence for a reduction in sea level of at least 80 m and by implication confirm the substantial estimates for the size of the ice sheets [57]. Smaller oscillations in relatively low sea level reported from Meifod may be local phenomena or may reflect eustatic changes that have now been described elsewhere [58,59]. This evidence speaks to a major ice age and in the intervening years; many studies [11,12,37] have confirmed this major Hirnantian regression.

The extinctions are coincident with and causally linked to this icehouse interval that has been proposed to be very short [23], with extinction intensity related to a lack of long north-south coastlines and the presence of abundant small islands and terranes [60].

**Figure 7.**
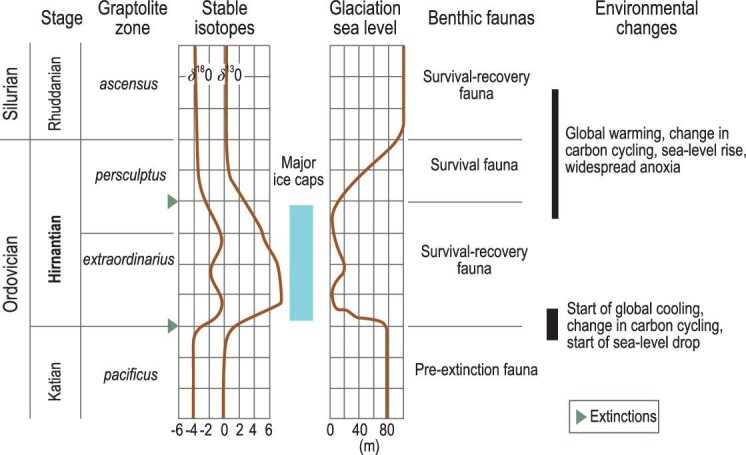
Stratigraphical window and biotic and environmental events through the Ordovician-Silurian boundary (redrawn from [57]).

Environmental changes have also been linked to stable isotope curves globally through Upper Ordovician successions. The Hirnantian isotope carbon excursion is the first developed and most widely used global chemostratigraphic marker in Ordovician successions, clearly illustrated in both δ^13^Ccarb and δ^13^Corg records [22,59]. The HICE is commonly used as a marker for the Hirnantian, reflecting a positive δ^13^C excursion (Fig. 7). There are two contrasting explanations for the origin of the Hirnantian δ^13^C excursion; the increased organic carbon burial [22] and the increased δ^13^C of river input from a weathered hinterland [61]. Both of these models result in parallel increases in δ^13^Ccarb and δ^13^Corg. However, a detailed study of Hirnantian successions on Anticosti Island notes that the observed relative timing between sea-level fall and the beginning of the δ^13^C excursion is consistent with the weathering hypothesis and provides an explanation for the origin of the Hirnantian positive δ^13^Ccarb excursion [61,62]. The excursion was reported to persist through the deglacial sea-level rise, indicating that the Anticosti strata records the response time for the Hirnantian carbon cycle perturbation [62].

Plate tectonics has been responsible for global mega environmental changes. Widely-discussed in literature is the drift of the large southern continent, Gondwana, over the South Pole, causing the onset and occurrence of a brief but extensive glaciation. The subsequent accumulation of ice mass was documented to extend across Gondwana and adjacent terranes, cooling the southern oceans and lowering sea levels globally due to the locked water in the ice. This tectonic-driven paleotemperature drop enabled the polar faunas to migrate towards the tropics, but many warm-water faunas in the tropical belt could not adapt and died out. Following this short-term glaciation, ice sheets began to melt, leading to rising sea levels, global heating and widespread marine euxinia, which finally initiated the second phase of extinction during the Late Ordovician. Environmental change, particularly the spread of carbonate shelves during the Silurian, favoured, within the Brachiopoda, the atrypids, athyridids, pentamerids and spiriferids, as the more typical Ordovician communities of orthides and strophomenides were in gradual decline [63,64]. Reef build-ups with corals and stromatoporoids were obvious components of communities at lower latitudes [65].

## ECOLOGICAL CHANGES

The marine life in the entire Phanerozoic is commonly described in terms of three great evolutionary faunas [66]. They include the Cambrian Evolutionary Fauna dominated by the morphologically-variable trilobites, and early brachiopods, echinoderms and molluscs, in combination with loosely-structured communities, the Palaeozoic Evolutionary Fauna covering the periods from Ordovician to Permian with the dominance of suspension feeders of benthos for example brachiopods, bryozoans and corals in well-structured communities, and the Modern Evolutionary Fauna spanning the periods from Triassic to the Recent featured by the dominance of deposit feeders, in a well-documented, escalating arms race between predators and prey.

The GOBE [34,67], witnessed an unparalleled radiation of marine life that included elements of the Palaeozoic Evolutionary Fauna with surviving taxa from the Cambrian Evolutionary Fauna with, already, components of the Modern Evolutionary Fauna. During the Hirnantian ∼45% of the Cambrian Evolutionary Fauna disappeared, together with 30% and 5% of the Paleozoic and Modern evolutionary faunas, respectively. The extinction marks the end of the Ecological Evolutionary Unit EEU P2, mainly occupied by the diversifications within the envelope of the GOBE [11]. Post-extinction, members of the suspension-feeding Paleozoic Evolutionary Fauna spread across the planet's sea beds whereas the detritus-feeders of the Modern Evolutionary Fauna continued to radiate, mainly in near-shore environments.

Although the LOME marked a substantial taxonomic extinction, in terms of an ecological event its significance has been downgraded. The ecology of the Ordovician radiation and the extinctions at the end of the period have been highlighted in a number of studies [68,69]. In the most recent survey of the ecological severity of the 11 largest Phanerozoic diversity crises [70,71] the Hirnantian is rated seventh equal with the Famennian (whereas it is third in taxonomic severity). Ecological changes occurred predominantly within Bambachian megaguilds, reflecting level 3 changes, as taxa were displaced and replaced in phases of expropriations [72].

**Figure 8.**
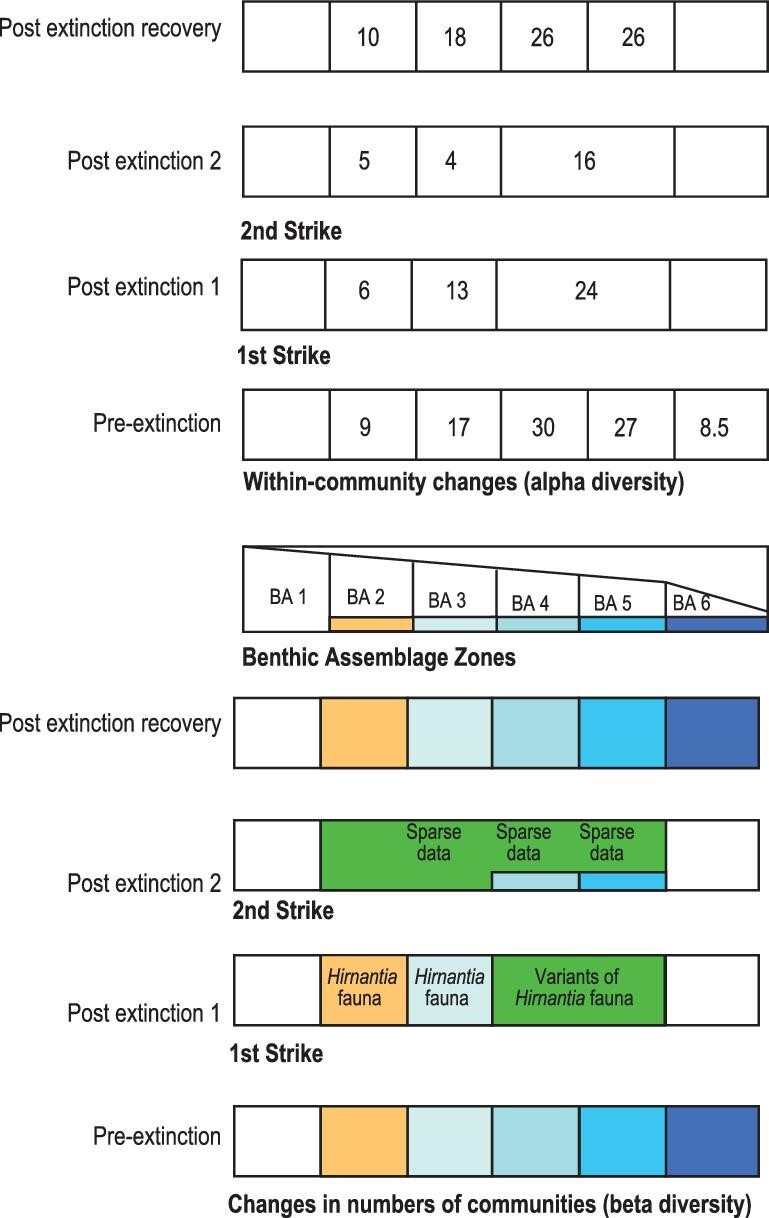
Changes in the alpha (upper) and beta (lower) diversity profiles through the extinctions. Changes in alpha diversity are based on the average species richness of communities. There are no data for BA 1. For beta diversity, distinct communities are shown by the colour of that zone. If a community occurs in two or more zones, a separate colour is used (replotted from [25]).

During the extinction events, a number of clear biogeographic and ecologic patterns have emerged. Brenchley *et al.* [25] have investigated the loss in alpha (within community) and beta (between community) biodiversity in individual communities and the numbers of communities that were lost during the Hirnantian Age (Fig. 8). In the first pulse there was a slight diminution of taxa in mid-shelf to outer shelf settings, despite an overall substantial disappearance of brachiopod taxa. The deep-water *Foliomena* fauna vanished though risk analysis, based on its previous longevity, did not predict its demise [73]. The origination and invasion of the *Hirnantia* brachiopod fauna [28,31], into high to temperate latitudes, helped rejuvenate some early Hirnantian assemblages to levels near to those at the end of the Katian. But the brachiopod taxa inhabiting the outer shelf and upper slope environments, including the *Foliomena* fauna, were completely wiped out. Notably, however, a substantial part of the Brachiopoda inhabiting the mid- and outer shelf, including the widespread *Hirnantia* brachiopod fauna, was eradicated during the second pulse [29,30,32].

A reef gap was observed during the Hirnantian just as seen in some other critical periods such as the early Triassic after the end-Permian crisis, indicating this key ecosystem all but disappeared during the biotic crisis in Earth history. Reef ecosystems are documented to suffer from a high risk of extinction and in particular require time to recover when subjected to cool temperatures and acidity. Reefs were prominent again during the Llandovery of the early Silurian with taxonomic compositions similar to those of the Katian of the Late Ordovician; these reefs continue to show a dominance in many Silurian carbonate environments after their revival. The build-ups of the reef ecosystems were featured by the dominance of the same families of stromatoporoids and tabulate corals, although the latter suffered substantial losses. A number of taxa, having disappeared during the extinction, were found only to reappear during recovery intervals after the main biotic crisis. Thus, the Silurian reef faunas may be interpreted as Lazarus taxa and the reefs, a Lazarus ecosystem.

The Lilliput Effect on the body size change of animals has been documented to show a relation to the survival in the deteriorated environmental conditions after some major catastrophes, particularly during the well-known end-Permian crises [6]. However, Brachiopoda reacted differently in terms of taxonomic level. For example, orthide brachiopods within surviving lineages increased their body sizes in the extinction horizons and subsequent recovery, followed by significant harm after the crisis [74]. In contrast, surviving pentameride and rhynchonellide brachiopods radiated rapidly during the earliest Silurian in association with a decrease in their body size. These differing Lilliput effects seen in these two major groups showed their discrimination of survival strategies, with the latter two showing the more successful trajectory [74].

**Figure 9.**
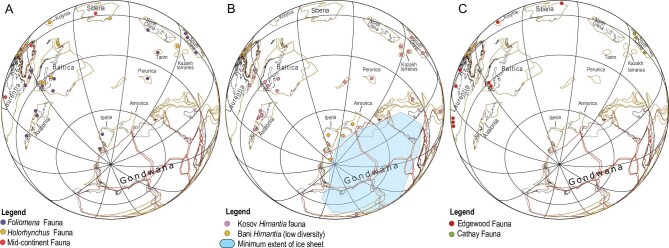
Palaeogeographic distributions of the main brachiopod faunas during the Late Ordovician: (A) Katian; (B) early Hirnantian; (C) late Hirnantian (figures courtesy of Dr. Christian Rasmussen). The circles are indicative of the general positions of the brachiopod faunas. For more accurate and detailed palaeogeographic maps see [32].

Provinces and bioregions are combinations of palaeocommunities; their numbers and those of individual taxa affect global or gamma biodiversity levels; Katian, early Hirnantian and late Hirnantian biogeographies of Hirnantian brachiopod bioregions are outlined, schematically (Fig. 9A–C). A number of authors have analyzed the changes in provinces through the event. For example, Sheehan and Coorough [75] noted that the number of bioregions changed significantly during and after the event, from some 10 in the Katian, to nine in the Hirnantian and only five in the early to middle Silurian. Following the first pulse there were sufficient endemics to characterize nine out of the 10 previous bioregions, although only three provinces were recognized with any clarity in the Hirnantian [29] and [76]; the assignment of the Edgewood bioregion is now correlated with slightly higher levels in the Hirnantian and in fact reduces the number of provinces to two [32]. The process of continental collision and amalgamation, further reduces the provinciality of faunas.

These combined biogeographical and ecological analyses suggest that losses occurred mainly within communities, as a reduction in alpha biodiversity [25,77], but had major influences on both beta and gamma diversity levels. Since the causes and severity of the two phases were in contrast, those taxa that survived the first wave of extinction commonly succumbed to the second. The first strike signals the destruction of selected habitats whereas the second was more extensive and pervasive.

## ONE CAUSE TO BIND THEM ALL?

**Figure 10.**
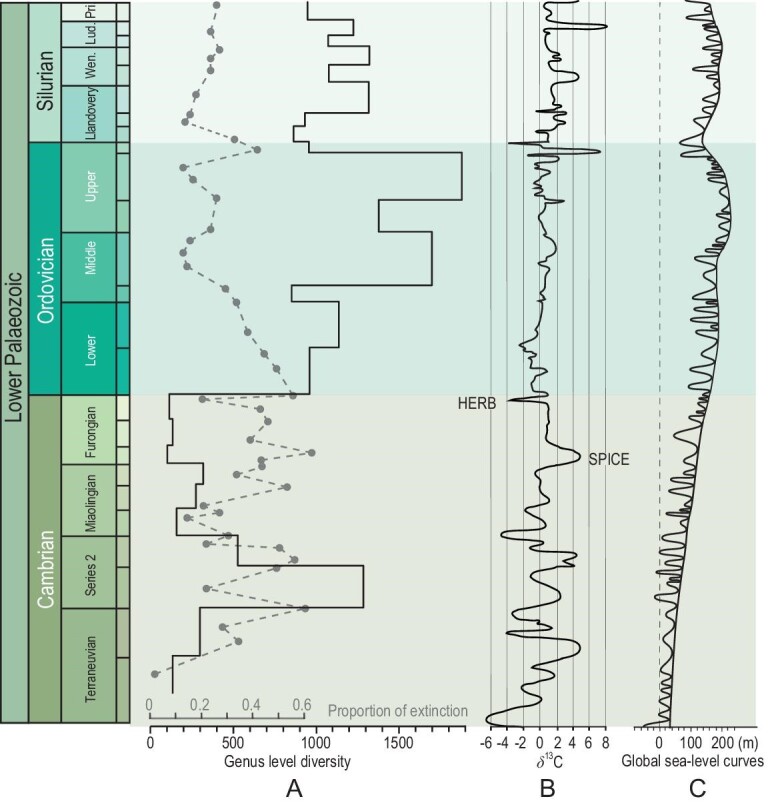
Global (data from Paleobiology Database: PBDB) marine genus-level diversity patterns (A) plotted against carbon isotopes (B), together with the global sea-level curve (C) (from [38]; isotope and sea level curves from [33]).

The terminal Ordovician ice age is one of a series of Ordovician glaciations identified on the basis of changing bathymetry [78], increasing in intensity and global reach since the Tremadocian (early Ordovician). During the later Ordovician, global cooling had already initiated the establishment of ice sheets on polar Gondwana during the Katian or even earlier [1,78]. A number of studies have associated sea-level changes, stable isotope curves, temperature, and generic and species richness data [32,79], many graphs combine these data [33,38; Fig. 10].

The LOME has been related to a variety of coincident or closely sequential changes in environmental conditions which drove the organisms to migrate, adapt and evolve or to become extinct. The consequences of the spread of the ice mass in Gondwana land were significant just as observed in the first pulse (Fig. 8), including a glacioeustatic sea-level drop of some 100 m [21], decrease in global temperatures of at least 5°C [79], and the anoxic conditions leading to poisonous environments creeping up over the outer shelves [31]. The reduction in the numbers of terranes [36], the lack of extensive cross-latitudinal coastlines and a diversification of small island complexes may have further accelerated the biotic crisis and could be contributory factors [60]. The second extinction pulse occurred when the glaciers receded in Gondwana and the anoxic seawater encroached the shelves flooded by a more stagnant water column in the scenario of warmed oceans.

**Figure 11.**
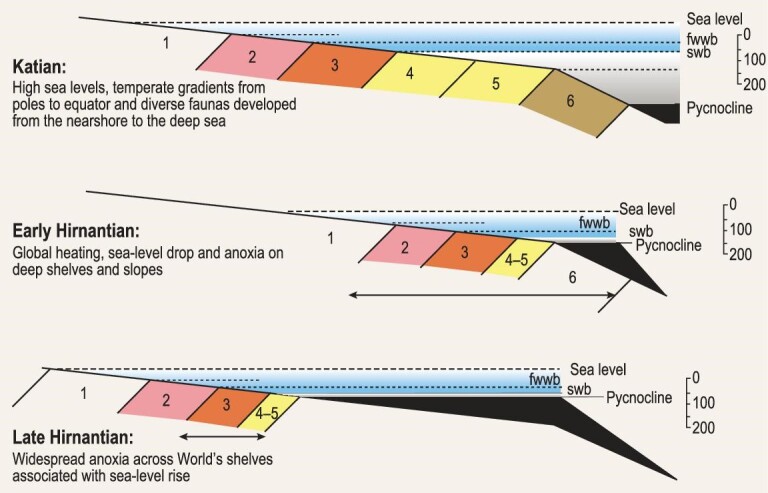
A simplified block model for the end Ordovician extinctions, invoking fluctuating temperatures, sea levels and anoxia, the three main environmental determinants (modified from [82]).

The LOME has been modeled, highlighting the comprehensive effects of reduced water temperature, sea-level drop and anoxic water encroachment onto the upper slope from the deep shelf, supported by biotic, isotopic and sedimentological data [80–82; Fig. 11]. However, no clear consensus was reached concerning the overriding cause to initiate and sustain this very short-lived, intense and damaging glaciation. Consequently, the primary cause (trigger) of the causes (killer) to the biotic crisis remains largely elusive though evidence is mounting for major, albeit cryptic, volcanic activities during the pre- and contemporary Hirnantian Stage.

Nevertheless, a plethora of potential causes have been cited [11,12,36]. The majority are associated with major, environmental swings but three involved possible biological drivers:

- Extensive volcanic eruptions may have already changed the climate before the Hirnantian by direct forcing, creating an increased albedo assisted by ash particles in the atmosphere and the weathering of volcanic rocks [83]. Such atmospheric effects, however, usually act over at most a decade although the eruptions may have been sustained and their products may have reached the stratosphere. The major volcanic eruptions of this interval, however, apparently occurred during the Sandbian Stage, when there were widespread tephra falls. But the after-burn of these may have influenced the transition towards cooler climates during the later part of the Late Ordovician. New studies, however, have revived interest in volcanicity as a causal factor for the terminal Ordovician glaciation, the idea of a volcanic trigger first introduced over a decade ago [83–85]. The volcanic eruptions were documented by spikes in the mercury enrichment of sediments in different locations of the world during the biotic crisis of the Hirnantian Stage or just below the Katian-Hirnantian boundary, including the Laurentia and South China [86], and the Holy Cross Mountains on the southern Baltic margins [87]. Hirnantian volcanics and volcaniclastics, probably related to a Large Igneous Province (LIP) have been recently reported from the Alborz Mountains in northern Iran [88] in addition to ash beds already reported from South China [23]. Support for a longer more sustained phase of volcanicity has been presented more recently, suggesting volcanoes were active some 10 myr prior to the Hirnantian Age [1].
- A hypothesis involving the rate of silicate weathering in controlling levels of atmospheric CO_2_ requires the exposure of silicates to enhance weathering [89]; the transit of silicate-dominated massifs through the intertropical convergence zone where high rainfall would have favoured chemical weathering as would terrestrial vegetation. The ensuing chemical reaction created bicarbonate ions that acted as a sink for carbon. Basalts, when exposed to the atmosphere, were especially reactive providing a link with the coeval volcanicity during the Late Ordovician Epoch. As more land area became exposed as the regression proceeded, chemical weathering escalated and intensified, although terranes at higher latitudes probably remained ice bound.
- Additional tectonic mechanisms have been posited that include the drift of the Gondwana supercontinent over the South Pole, causing an additional albedo effect via the land-based ice and snow. Furthermore, the Taconic Basin, formed on the edge of ancient Laurentia during the post-Caledonian extension, may have induced the diversion of cooler currents along the marine margins [90].
- Metal pollution signaled by mercury enrichment and other heavy metals suggest pollution by toxins of the Late Ordovician oceans; this may be due to the direct input of volcanic material or hydrothermal activity. Heavy metal pollution has been illustrated by malformations in fossil plankton [91].
- The Monterey Hypothesis posits that in deep-sea canyons off the Californian coast, the increased primary production prior to glaciation during the Miocene, in particular in the regions featured by the occurrence of upwelling, further stimulated the enhanced carbon burial to draw down the levels of atmospheric CO_2_ [92]. However, evidence for such a scenario with widespread upwelling causing extensive carbon burial in deep-sea sediments, or even for a concomitant decrease in the atmospheric CO_2_ level, is sparse during the latest Ordovician [82].
- Blooms of algae and other elements of the phytoplankton may have also responded to the influx of nutrients from volcanic activity and increased weathering of land areas, particularly those exposing silicate rocks. This biological activity provided excessive amounts of the lighter carbon isotopes for burial, thus driving positive excursion while capturing CO_2_ and cooling of the planet [21,93].
- The diversification of land plants has been posited to explain the rapid climate cooling required to initiate the extensive glaciation [94]. The new Ordovician terrestrial flora may have accelerated the silicate weathering which in turn enhanced the release of key ions from rock-derived minerals into the oceans, especially phosphorous. These terrestrial nutrient inputs inspired the blooms of marine autotrophs which assisted the carbon burial and enhanced drawdown of atmospheric CO_2_; ironically, however, the early evolution and radiation of land plants was proposed as a driver of the marine biotic crisis of the latest Ordovician [94].

## AN EARLIER LATE ORDOVICIAN EXTINCTION

**Figure 12.**
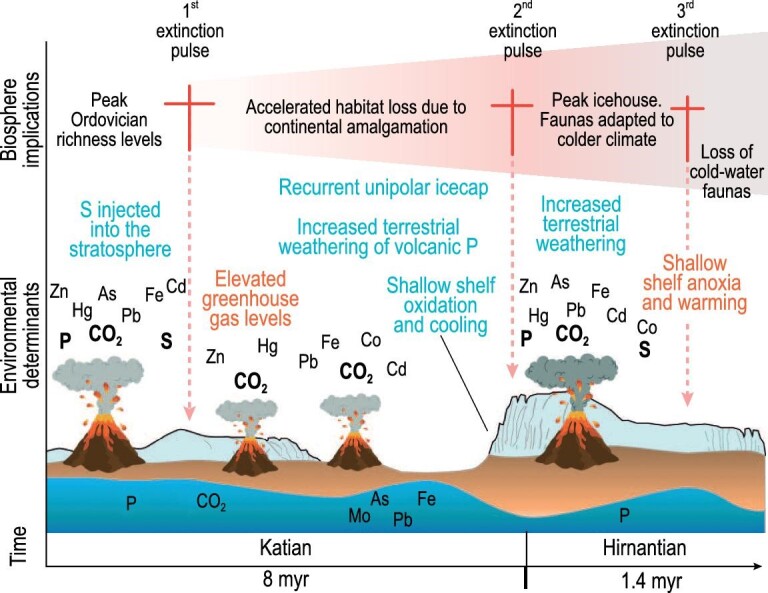
A revised terminal Ordovician scenario with three Late Ordovician mass extinctions. Late Ordovician mass extinctions (LOME) indicating pollution of the ambient environment; additionally habitats were destroyed through natural continent–continent collisions during the extinctions and survival phase of LOME (from [1]: figure courtesy of Dr Christian Rasmussen).

In addition to much new data, both biological and geochemical, recent temporally better-resolved fossil biodiversity estimates are providing growing evidence for a prolonged but nevertheless punctuated biodiversity decline driven by changes in atmospheric composition, ocean chemistry, and habitable area [1]. This developing scenario invokes overarching extinction drivers similar to those that occurred during other major extinctions [85] and focuses on extended and profuse volcanic activity (Fig. 12). The extinction window was, however, much wider than previously considered extending back to the early Katian and possibly the Sandbian. The longevity of the event, some 10 myr, aligns with that of other major extinction events but, nevertheless, the key pulse was short and sharp. Rasmussen *et al.* [1] noted the relevance of the LOME, its drivers and diversity to the current environmental crisis whereby anthropogenic impacts operate in much the same way as volcanicity in changing conditions on the planet, its biotas and the composition of its atmosphere [1].

## SIGNIFICANCE OF THE LOME

The LOME is the initial mass extinction that affected marine metazoans and had an important influence on the subsequent trajectory of marine life on the planet. During mass extinctions ecosystems and habitats may be entirely or just partially destroyed but life survives and rebounds. The end Ordovician extinctions were the first major test of this hypothesis in global marine biotas and although displaying a major two-phase taxonomic extinction, the ecological and phylogenetic dimensions of the events were less pronounced. Destruction of habitats and the extinction of lower taxa were marked but so too was the survival and recovery. Microbes capitalized first in some environments [44] then the shelves, slopes and finally the deep sea slowly recovered, onshore–offshore communities were repopulated, retaining the same overall structures but were commonly infilled with different taxa from those in Ordovician communities. The analogy of refurnishing a house is apt. Old furniture is often discarded in favour of new more fashionable and stylish items. Nevertheless, the new furniture still fulfils the key functions and roles within the house of the discarded beds, chairs and tables. For example, within the Brachiopoda groups such as the orthide-strophomenide plexus that dominated communities in the Ordovician were gradually replaced in the Silurian by those with atrypides, athyridides, pentamerides, rhynchonellides and spiriferides, initially in low-latitude carbonate environments in particular [32,63]; the large pentamerides and nonarticulated trimerellides formed extensive local shell banks. Changes occurred within communities perpetuating the dominance of suspension-feeders until the terminal Permian extinction [95]. More importantly, the extinction events may have cleared the ground for the diversification and expansion of the fishes in the oceans as they occupied the seabed and moved into the water column during the Nekton Revolution [55]; life on land, both animal and plant taxa, prospered [96].

## CONCLUSIONS

1. The extended LOME was at least 1.4 myr in duration, the main Hirnantian phase was only ∼200 kyr, and new data suggest there may have been a third phase in the early Katian stretching the extinction window to ∼10 myr. There were two phases in the Hirnantian and possibly an earlier phase in the early Katian.
2. Most groups of micro- and macro fauna were affected with the benthos most hard hit. It was a significant taxonomic event but less of a phylogenetic or ecological crisis.
3. The main agents of environmental changes globally and regionally that facilitated or triggered habitat destruction or restriction, biotic migrations and eventual extinction involved a major fall in sea level, global cooling and the encroachment of the slopes and deep shelves by anoxic bottom waters.
4. The overarching cause or causes of these environmental changes were apparently related to long-term volcanism and its eruptive products, together with the impact of its weathered residues.
5. The taxonomic extinctions changed the composition of marine communities and probably helped prepare marine environments for the Nekton revolution and, on land, more extensive terrestrialization.
